# Modeling absolute differences in life expectancy with a censored skew-normal regression approach

**DOI:** 10.7717/peerj.1162

**Published:** 2015-08-06

**Authors:** André Moser, Kerri Clough-Gorr, Marcel Zwahlen

**Affiliations:** 1 Department of Geriatrics, Bern University Hospital, and Spital Netz Bern Ziegler, and University of Bern, Bern, Switzerland; 2 Institute of Social and Preventive Medicine (ISPM), University of Bern, Bern, Switzerland

**Keywords:** Life expectancy, Skew-normal regression, Left-truncation, Survival regression, Censoring

## Abstract

Parameter estimates from commonly used multivariable parametric survival regression models do not directly quantify differences in years of life expectancy. Gaussian linear regression models give results in terms of absolute mean differences, but are not appropriate in modeling life expectancy, because in many situations time to death has a negative skewed distribution. A regression approach using a skew-normal distribution would be an alternative to parametric survival models in the modeling of life expectancy, because parameter estimates can be interpreted in terms of survival time differences while allowing for skewness of the distribution. In this paper we show how to use the skew-normal regression so that censored and left-truncated observations are accounted for. With this we model differences in life expectancy using data from the Swiss National Cohort Study and from official life expectancy estimates and compare the results with those derived from commonly used survival regression models. We conclude that a censored skew-normal survival regression approach for left-truncated observations can be used to model differences in life expectancy across covariates of interest.

## Introduction

Absolute differences in life expectancy are of importance in many scientific fields, i.e., biology, demography and epidemiology (Aldenhoven, 1986; Liu et al., 2012; Olshansky et al., 2012; Sarkar et al., 2010; Spoerri et al., 2006). Often, differences in life expectancy are calculated by traditional life table methods (Chiang, 1984) using sex and age specific mortality rates. Additional covariate information (e.g., education or marital status) and corresponding death rates are usually not available, but absolute differences across levels of such covariates are of interest. Nationwide census based cohort studies (Bopp et al., 2009; Frisch & Simonsen, 2013; Spoerri et al., 2010; Ueda et al., 2013) allow the investigation of mortality trends and the calculation of life expectancy conditional on covariates on individual, household and area level. In such large cohort studies, demographers and public health researchers are interested in exploring joint associations of several covariates on individuals’ lifespan, and to quantify absolute differences in life expectancy. Parametric survival regression is a common method to model covariate effects on survival time by either using proportional hazard models or accelerated failure time models (Harrell, 2001). The first type of survival model reports covariate effects in terms of hazard ratios, the latter in terms of time ratios. However, both effect measures cannot be directly interpreted in terms of differences of life expectancy, since they are on a transformed scale. For example, an estimated hazard ratio of two for men compared to women from a proportional hazard model yields no direct interpretation in terms of life expectancy differences, say, that men live on average five year less long than women. A method for converting hazard ratios from a Cox proportional hazard model to life expectancy has been developed, but is rather cumbersome and complex in its implementation (Fontaine et al., 2003; Robertson, De Los Campos & Allison, 2013).

Gaussian linear regression models are commonly used for data analysis in many scientific disciplines (e.g., in biology, medicine, psychology, agriculture) with the advantage that estimated regression parameters are easily interpreted in terms of mean differences in the outcome per one unit change of the predictor variable. Nevertheless it is well known that departures from the underlying model assumptions (i.e., residuals have to follow a Gaussian distribution with constant variance) lead to biased results and inappropriate interpretations (Harrell, 2001). If residuals from a Gaussian regression are negative skewed, data transformations are required to fulfil the desired underlying model assumptions with the drawbacks that such transformation functions are often not available, or, if they exist, the regression results are also on the transformed scale and are more difficult to interpret. Skew-normal distribution functions extend the class of Gaussian distribution functions by an additional shape parameter which allows for skewness in the distribution. The class of skew-normal distribution functions has been comprehensively investigated by Azzalini in the 1980s and subsequently extended (Azzalini & Capitanio, 1999; Azzalini & Capitanio, 2003; Azzalini & Dalla Valle, 1996); for a broad overview see the books of Genton (2004) or Azzalini & Capitanio (2014).

Gaussian-related distribution functions have been shown to be a possible alternative to commonly used generalized extreme value distributions (e.g., Gompertz distribution) in the modeling of life expectancy (Clark et al., 2013; Robertson & Allison, 2012; Robertson, De Los Campos & Allison, 2013). Clark and colleagues (2013) found that a skew-*t* distribution outperformed a Gompertz-like distribution function in modeling mortality data in terms of model fit. Robertson & Allison (2012) evaluated a compressed Gaussian distribution in the modeling of human longevity. The authors confirmed the findings of Kannisto (2001) that the distribution of longevity conditioned on survival to the modal age was similar to a Gaussian distribution. Robertson and colleagues (2013) used a censored regression approach for left-truncated data with an underlying compressed Gaussian distribution function in modeling life expectancy. The authors found that median differences of life expectancy were similar from Gaussian-type regression models and others.

To our knowledge, no implementation is currently available to use a skew-normal modeling approach in survival analysis situations in which a fraction of the observations have censored survival times and in which delayed entry is present. Delayed entry occurs e.g., in situations in which subjects are observed from a study entry date until end of the study, and not over the total risk time from a certain given age. In this article we describe how we implemented censored skew-normal regression models for survival data, give results when analyzing life expectancy for the Swiss population using data from the Swiss National Cohort Study and official estimates of life expectancy in Switzerland.

## Methods

### Data

The Swiss National Cohort (SNC) is a longitudinal study of the entire resident population of Switzerland, based on the 1990 and 2000 national censuses (Bopp et al., 2009). Deterministic and probabilistic record linkage (Fellegi & Sunter, 1969) were performed using the Generalized Record Linkage System (Fair, 2004) to link census records to a death record or an emigration record, based on a set of key variables that are available in both data sets (sex, date of birth, place of residence, marital status, religion, nationality, profession, date of birth of partner and date of birth of children). Mortality patterns and life expectancy patterns are of major interest in the SNC (Moser et al., 2014; Spoerri et al., 2014; Spoerri et al., 2006). Initial SNC mortality linkage was successful for 94% of the deaths (Bopp et al., 2009). The 6% not linked deaths bias the calculation of absolute age-specific mortality rates which in turn would bias estimates of life expectancy (Schmidlin et al., 2013). This bias was removed when including the 6% deaths using pragmatic linkages matching for age and sex only. For analyses presented here, we used the SNC data from the 2000 census onwards with mortality follow-up up to end of 2008 including the pragmatically linked deaths. We investigated 4,098,675 individuals aged ≥35 years or older from the 2000 census. Of those, 481,157 individuals died between 5th December 2000 (date of census) and 31th December 2008. We investigated associations of gender, civil status and education on individual’s life span, using parametric survival regression and censored skew-normal and censored Gaussian regression approaches.

For a second analysis and the simulation study we used data from the Human Mortality Database (HMD) which contains death rates and life tables from various populations including Switzerland (Human Mortality Database, 2014). Data are provided from national statistical offices or other sources. We used death rates for one year age intervals from age 35 to 105. For a hypothetical population of *N* = 100, 000 men and women we estimated the number of deaths for each one year age interval from age 35 to age 105, *I*_[*x*,*x*+1]_ ≔ *I_x_*, *x* ∈ 35, …, 105, as follows. The death rate for an age interval *I_x_*, *x* ∈ 35, …, 105, is denoted as *m_x_*. The number of persons alive at the start of the age interval *I_x_* is denoted as *l_x_*, *x* ∈ 36, …, 105. *l_x_* is equal to 100,000 minus all the deaths in all age intervals before *I_x_*. The number of deaths *n_x_* for each one year age interval *I_x_* was then calculated as *n_x_* = *m_x_* ⋅ *l_x_*, *x* ∈ 35, …, 105. Data were downloaded and analyzed using the R-package demography (Hyndman, 2012).

### Parametric survival models

Parametric survival regression assumes that, for each individual, the time *T* elapsed from a starting time point (e.g., at age 35) to an event (e.g., death) has a cumulative distribution function (cdf) *F*(*t*) and a probability density function (pdf) *f*(*t*) from certain classes of distribution functions. Often one assumes a distribution function from an extreme value type, e.g., Weibull, or from a lifetime type, e.g., Gompertz. The survival function is defined as *S*(*t*) = 1 − *F*(*t*) = ℙ(*T* > *t*).

One basic concept of survival regression is the hazard function, defined as

(1) \begin{eqnarray*} \displaystyle \lambda (t)=\lim _{u\rightarrow 0}\frac{\mathbb{P}(t\leq T\lt t+u\vert T\geq t)}{u}=\frac{f(t)}{1-F(t)}=\frac{f(t)}{S(t)}.&&\displaystyle \end{eqnarray*}

The survival function can then be expressed in terms of the hazard function as

\begin{eqnarray*} S(t)=\exp \left(-\int \nolimits _{0}^{t}\lambda (y)d y\right). \end{eqnarray*}

If one assumes that the observed survival time of each individual is a realization from the same distribution of *T* (without covariates representing differences between the groups of individuals) and *T* comes from a Weibull distribution function, then

\begin{eqnarray*} \lambda (t)=\frac{\gamma }{\alpha }{\left(\frac{t}{\alpha }\right)}^{\gamma -1},\hspace{1em}S(t)=\exp \left(-{\left(\frac{t}{\alpha }\right)}^{\gamma }\right),\hspace{1em}t> 0, \end{eqnarray*}

where *α* > 0 is a scale parameter and *γ* ≥ 0 a shape parameter of the Weibull distribution, or when *T* is Gompertz distributed with scale parameter *α* > 0 and shape parameter *γ* > 0, then

\begin{eqnarray*} \lambda (t)=\alpha \exp \left(\gamma t\right),\hspace{1em}S(t)=\exp \left(-\frac{\alpha }{\gamma }\left[\exp (\gamma t)-1\right]\right),\hspace{1em}t> 0. \end{eqnarray*}

For the survival regression problem one often assumes that for a set of covariates **X** = (*X*_1_, …, *X_k_*)^⊤^ the following equation holds

\begin{eqnarray*} \lambda (t\vert \mathbf{X})=\lambda (t)\exp \left({\mathbf{X}}^{\top }\boldsymbol{\beta}\right), \end{eqnarray*}

where **β** = (*β*_1_, …, *β_k_*)^⊤^ is a vector of regression coefficients. This model specification is known as *proportional hazard* (PH) model and *λ*(*t*) is then often called an *underlying baseline hazard function*. In case that *T* is assumed to be Weibull distributed one gets

\begin{eqnarray*} \lambda (t\vert \mathbf{X})=\frac{\gamma }{\alpha }{\left(\frac{t}{\alpha }\right)}^{\gamma -1}\exp \left({\mathbf{X}}^{\top }\boldsymbol{\beta}\right), \end{eqnarray*}

or with the assumption of a Gompertz distribution

\begin{eqnarray*} \lambda (t\vert \mathbf{X})=\alpha \exp \left(\gamma t\right)\exp \left({\mathbf{X}}^{\top }\boldsymbol{\beta}\right). \end{eqnarray*}

Note that in such a model representation the relationship between the covariates and the hazard function are linear on the log hazard scale. Thus, the effect of increasing a continuous covariate, say *X*_1_, by *d*, holding all other variables constant, is to *increase the hazard of the event* by a factor of exp(*β*_1_*d*) at all time point in time, assuming *X*_1_ is linearly related to log*λ*(*t*) (Harrell, 2001). Often the above described effect is reported in terms of hazard ratios, i.e., one compares the ratio of hazard rates of an individual with predictor value *d* compared to one with a value of 0. The hazard ratio is then $\lambda (t)\exp \left({\beta }_{1}d\right)/\lambda (t)=\exp \left({\beta }_{1}d\right)$. Unlike the interpretation of a multivariable linear Gaussian regression model, where the regression coefficients **β** are reflecting an increment in the expected value of a response variable by a one unit change in the predictor variable, the interpretation of regression coefficients from survival regression models is not easily translated to differences in mean survival time.

Another often used type of survival model is the *accelerated failure time* (AFT) model (see e.g., Harrell (2001)), where one assumes that

(2) \begin{eqnarray*} \displaystyle \log (T)={\mathbf{X}}^{\top }\boldsymbol{\beta}+\sigma \epsilon ,&&\displaystyle \end{eqnarray*}

with *σ*, a scale parameter, and *ϵ* is assumed to come from a survival distribution function *ϑ*.

Common choices of *ϑ*(*u*) are the logistic distribution *ϑ*(*u*) = [1 + exp(*u*)]^−1^ (log-logistic model) or the Gaussian distribution *ϑ*(*u*) = 1 − Φ(*u*) (log-normal model). Both distributions fail to fit lifetime data adequately, because of their positive skewed distribution. To address a negative skewed distribution, an underlying Weibull distribution is possible. Note from (2) that in the AFT model specification the interpretation of the estimated parameters are in terms of *T* = exp(**X**^⊤^**β** + *σϵ*). The effect of increasing a continuous covariate, say *X*_1_, by *d*, holding all other variables constant, is to *increase the survival time* *T* by a factor of exp(*β*_1_*d*), assuming Eq. (2). Similarly to reporting hazard ratios in PH models, one reports time ratios in AFT models, i.e., the ratio of survival times of an individual with predictor value *d* compared to one with a value of 0. The time ratio is then exp(*β*_1_*d* + *σϵ*)/exp(*σϵ*) = exp(*β*_1_*d*). Also in this type of survival modeling, interpretation of regression coefficients is not straightforward in terms of mean survival time. Note that the Weibull PH model and the Weibull AFT are equivalent (Harrell, 2001).

### Life expectancy from survival models

It is well known that life expectancy (or expected survival time) conditional on covariates 𝔼(*T*|**X**) is related to the conditional survival function *S*(*t*|**X**) through

\begin{eqnarray*} \displaystyle \mathbb{E}(T\vert \mathbf{X})=\int \nolimits _{0}^{\infty }S(t\vert \mathbf{X})d t=\int \nolimits _{0}^{\infty }{\left\{S(t)\right\}}^{\exp ({\mathbf{X}}^{\top }\boldsymbol{\beta})}d t,&&\displaystyle \end{eqnarray*}

where *S*(*t*) is the underlying survival distribution, see e.g., Harrell (2001). Hence, the expected survival time for a reference individual is $\mathbb{E}(T\vert \mathbf{X})=\int \nolimits _{0}^{\infty }S(t)d t$. For Weibull, Gompertz and log-normal distribution functions closed-form expressions exist, i.e., for the Weibull distribution

\begin{eqnarray*} \mathbb{E}(T\vert \mathbf{X})=\alpha \Gamma (1+1/\gamma ), \end{eqnarray*}

for the Gompertz distribution

\begin{eqnarray*} \mathbb{E}(T\vert \mathbf{X})=\frac{1}{\gamma }\exp \frac{\alpha }{\gamma }\int \nolimits _{\alpha /\gamma }^{\infty }{x}^{-1}\exp (-x)d x, \end{eqnarray*}

and for the log-normal distribution

\begin{eqnarray*} \mathbb{E}(T\vert \mathbf{X})=\exp (\mu +{\alpha }^{2}/2), \end{eqnarray*}

where *μ*, *α*, *γ* are location, scale and shape parameters from the corresponding distribution functions, see e.g., Johnson, Kotz & Balakrishnan (1994) and Missov & Lenart (2011). 95% confidence intervals (CI) were approximated by sampling procedures from multivariate Gaussian vectors using the covariance matrices of the parameter estimates.

### Censored skew-normal regression

The skew-normal distribution function generalizes the Gaussian distribution function allowing for skewness in its shape. We start with a recapitulation of the definition of a Gaussian distributed random variable. A random variable *X* is Gaussian (normal) distributed with location parameter *μ* ∈ ℝ and scale parameter *σ* > 0 if it has the pdf

\begin{eqnarray*} f(x;\mu ,\sigma )=\frac{1}{\sigma \sqrt{2 \pi }}{\exp }^{-\frac{1}{2}{\left(\frac{x-\mu }{\sigma }\right)}^{2}}. \end{eqnarray*}

One writes *X* ∼ *N*(*μ*, *σ*^2^) if *X* is Gaussian distributed with mean *μ* and variance *σ*^2^. The pdf of standard Gaussian distributed random variable *Z* ∼ *N*(0, 1) is in the following written as *φ*(⋅).

The definition of a skew-normal distributed random variable is as follows, see e.g., Genton (2004).

Definition 1 *A random variableYis skew-normal distributed with location parameterξ* ∈ ℝ*, scale parameter* *σ* > 0 *and shape parameter* *ψ* ∈ ℝ*, if it has the pdf*

(3) \begin{eqnarray*} \displaystyle g(y;\xi ,{\sigma }^{2},\psi )=\frac{2}{\sigma }\varphi \left(\frac{y-\xi }{\sigma }\right)\Phi \left(\psi \frac{y-\xi }{\sigma }\right),\hspace{1em}-\infty \lt y\lt \infty ,&&\displaystyle \end{eqnarray*}

*where* Φ(⋅) *is the cdf of a* *N*(0, 1)*-distributed random variable. If* *Y* *is skew-normal distribution we write* *Y* ∼ *SN*(*ξ*, *σ*^2^, *ψ*)*.*

The expectation and variance of a skew-normal distributed random variable *Y* ∼ *SN*(*ξ*, *σ*^2^, *ψ*) is

(4) \begin{eqnarray*} \displaystyle \mathbb{E}(Y)=\xi +\sigma \sqrt{\frac{2}{\pi }}\frac{\psi }{\sqrt{1+{\psi }^{2}}}\hspace{1em}\text{and}\hspace{1em}\mathbb{V}(Y)={\sigma }^{2}\left(1-\frac{2}{\pi }\frac{{\psi }^{2}}{1+{\psi }^{2}}\right).&&\displaystyle \end{eqnarray*}

Note that if *X* ∼ *N*(0, 1) and *Y* ∼ *SN*(0, 1, 0), then *X* and *Y* are equally distributed. Since the parameters (*ξ*, *σ*, *ψ*) are directly involved in the pdf representation (3), one speaks of a *direct parametrization* (DP). Another representation is the so-called *centered parametrization* (CP) (Azzalini, 1985), where one uses a reparametrization of (3). In brief, one uses centered parameters (*μ*, *α*, *γ*) in the parametrization of the problem, where *γ* is a measure of skewness defined as

(5) \begin{eqnarray*} \displaystyle \gamma =\frac{1}{2}(4-\pi )\text{sign}(\psi ){\left(\frac{{\psi }^{2}}{\frac{\pi }{2}+\left(\frac{\pi }{2}-1\right){\psi }^{2}}\right)}^{3/2},&&\displaystyle \end{eqnarray*}

and *ψ* is the same as in (3) (Genton, 2004). One has the following relation

\begin{eqnarray*} \mathbb{E}(Y)=\left\{\begin{array}{ll} \displaystyle \xi +\sigma \sqrt{\frac{2}{\pi }}\frac{\psi }{\sqrt{1+{\psi }^{2}}}\hspace{1em}&\displaystyle (\text{DP})\\ \displaystyle \mu \hspace{1em}&\displaystyle (\text{CP}), \end{array}\right. \end{eqnarray*}

such that the location estimate from a CP corresponds to the expectation of skew-normal distributed random variable in a DP. In Supplemental Information 1 we explain the principles and the conversion of CP to DP, and vice versa.

The skew-normal regression problem is similar to Gaussian linear regression. One assumes for a set of covariates **X** = (*X*_1_, …, *X_k_*)^⊤^ and regression coefficients **β** = (*β*_1_, …, *β_k_*)^⊤^ that

\begin{eqnarray*} Y={\mathbf{X}}^{\top }\boldsymbol{\beta}+\epsilon , \end{eqnarray*}

where *ϵ* ∼ *SN*(0, *σ*^2^, *ψ*) and is assumed to be independent across individuals. The effect of increasing a continuous covariate, say *X*_1_, by *d*, holding all other variables constant, is to increase the survival time *T* by a shift of *β*_1_*d*. For the conversion of DP to CP (or vice versa) in the regression problem only the distributional parameters need to be transformed accordingly (Azzalini & Capitanio, 1999).

### Parameter estimation

Parameters for a censored skew-normal regression can be estimated by maximum likelihood estimation. Let the underlying measurement scale be individual’s age at end of study *Y_i_* or censoring *C_i_*, i.e., min{*Y_i_*, *C_i_*}. Censoring is assumed to be non-informative (i.e., censoring is independent of the underlying time scale), or from type I censoring (i.e., follow-up time ends at predetermined time). We write *δ_i_* = 𝕀(*Y_i_* ≤ *C_i_*) for the indicator variable for an observed death, where 𝕀 is the indicator function. Under censoring the likelihood is

(6) \begin{eqnarray*} \displaystyle L(\xi ,{\sigma }^{2},\psi )&=\prod _{i\leq n:~\delta _{i}=1}g({y}_{i};\xi ,{\sigma }^{2},\psi )\prod _{i\leq n:~\delta _{i}=0}S({c}_{i};\xi ,{\sigma }^{2},\psi )&\displaystyle \nonumber\\ \displaystyle &=\prod _{i\leq n:~\delta _{i}=1}\frac{2}{\sigma }\varphi \left(\frac{{y}_{i}-\xi }{\sigma }\right)\Phi \left(\psi \frac{{y}_{i}-\xi }{\sigma }\right)\prod _{i\leq n:~\delta _{i}=0}S({c}_{i};\xi ,{\sigma }^{2},\psi )&\displaystyle \nonumber\\ \displaystyle &=\prod _{i\leq n}{\left[\frac{2}{\sigma }\varphi \left(\frac{{y}_{i}-\xi }{\sigma }\right)\Phi \left(\psi \frac{{y}_{i}-\xi }{\sigma }\right)\right]}^{{\delta }_{i}}{\left[S({c}_{i};\xi ,{\sigma }^{2},\psi )\right]}^{1-{\delta }_{i}},&\displaystyle \end{eqnarray*}

where *g*(⋅; *ξ*, *σ*^2^, *ψ*) is the pdf given in (3) and *S*(⋅; *ξ*, *σ*^2^, *ψ*) is the survival function assuming *F*(⋅) = *SN*(⋅; *ξ*, *σ*^2^, *ψ*). Choosing the relevant time scale is a crucial decision in modeling survival times. Often not the total risk time starting from time origin (e.g., date of birth) is observed, but only the risk time from study entry (the date at which a person entered the study and came under observation) until the end of the study. This concept is called delayed entry or left-truncation. In this case one has to consider the conditional distribution of *Y_i_* given that *Y_i_* ≥ *D_i_*, where *D_i_* is a given time point or individual’s age at study entry. Note that the likelihood of an uncensored individual *i* ≤ *n* : *δ_i_* = 1 is then

\begin{eqnarray*} {g}^{\ast }({y}_{i};\xi ,\sigma ,\psi \vert {Y}_{i}\geq {D}_{i})=\frac{g({y}_{i};\xi ,{\sigma }^{2},\psi )}{S({D}_{i};\xi ,{\sigma }^{2},\psi \vert {Y}_{i}\geq {D}_{i})},~-\infty \lt {y}_{i}\lt \infty ,~i=1\leq n, \end{eqnarray*}

and for a censored individual *i* ≤ *n* : *δ_i_* = 0,

\begin{eqnarray*} {S}^{\ast }({c}_{i};\xi ,\sigma ,\psi \vert {Y}_{i}\geq {D}_{i})=\frac{S({c}_{i};\xi ,{\sigma }^{2},\psi )}{S({D}_{i};\xi ,{\sigma }^{2},\psi \vert {Y}_{i}\geq {D}_{i})},~-\infty \lt {c}_{i}\lt \infty ,~i=1\leq n. \end{eqnarray*}

To obtain the likelihood of all individuals one replaces in (6) *g*(*y_i_*; *ξ*, *σ*^2^, *ψ*) by *g*^∗^(*y_i_*; *ξ*, *σ*, *ψ*|*Y_i_* ≥ *D_i_*), and *S*(*c_i_*; *ξ*, *σ*, *ψ*) by *S*^∗^(*c_i_*; *ξ*, *σ*, *ψ*|*Y_i_* ≥ *D_i_*), respectively. It has been mentioned in e.g., (Azzalini, 1985) that maximizing the negative log likelihood of (6) has singularity problems if *ψ* = 0, and yield convergence problems in the MLE. To overcome this problem it has been suggested to use the CP approach, which removes the singularity at *ψ* = 0, and has further advantages in faster convergence and improved likelihood shape over the DP (see e.g., Azzalini (1985) and Azzalini & Capitanio (1999)). We used the CP for the numerical derivation of the estimates by MLE using R Version 3.1.1 and Stata Version 13.1. Program code and used functions are provided as Supplemental Information 1.

### Model assessment

For all types of survival models, the model should adequately fit the data in order to obtain correctly interpretable estimates and correct coverage of confidence intervals. For PH models the relationship between the covariates and the log hazard should be linear. Further, the covariates affect the underlying distribution of the time variable by adding **X**^⊤^**β** to the log hazard function. The effect of the covariates is assumed to be the same at all time points. For AFT models each covariate affects log(*T*) linearly. Further, the underlying variance *σ* in Eq. (2) is a constant, independent of the covariates (Harrell, 2001). Assessing the model fit of parametric survival models is often restricted to e.g., graphical assessment by stratified predictor levels or stratified Kaplan–Meier estimates of the distribution of residuals. For the skew-normal regression problem *Y* = **X**^⊤^**β** + *ϵ*, where *ϵ* ∼ *SN*(0, *σ*^2^, *ψ*), certain assumptions must be validated (Harrell, 2001): residuals should have no systematic trend in central tendency against any predictor, they should have the same dispersion and they should have a skew-normal distribution in the predictor-space. These assumptions can be checked by median and lower and upper quartiles of the residuals, stratified by intervals of the predicted outcome. Distributional model assumptions of a skew-normal regression can be visually checked by a comparison of quantiles of estimated residuals and quantiles from a theoretical skew-normal distribution in a quantile–quantile plot.

We graphically compared the model fit from a Gompertz model with a skew-normal model by a log-hazard plot. In general from Eq. (1) one obtains that log*λ*(*t*) = log*f*(*t*) − log*S*(*t*). Note that for a Gompertz distribution the log-hazard is linear in the time scale *t*, i.e., log*λ*(*t*) = log*α* + *γt*, *t* ≥ 0. Thus, any non-linearity in the log-hazard from a skew-normal model would indicate a deviation from the Gompertz model fit.

Goodness-of-fit using official life expectancy estimates was assessed by Pearson’s chi-squared test statistic ${X}^{2}=\sum _{i}({O}_{i}-{E}_{i})^{2}/{E}_{i}$ (Agresti, 2013), where *O_i_* denotes the observed number of deaths from offical estimates in one year age intervals. We calculated expected number of deaths *E_i_* for one year age intervals from the underlying regression models. A larger value of *X*^2^ indicates a greater difference of *O_i_* − *E_i_* (Agresti, 2013).

### Model setting

SNC data were analyzed by survival regression models with remaining age at 35 years as the underlying time scale and delayed entry date as the 5th of December 2000. Assumed underlying survival distribution function was either Weibull or Gompertz for a PH model, or Weibull and log-normal for an AFT model. Individuals were censored if they were alive after 31th December 2008. For a direct comparison using the censored skew-normal regression approach we investigated age at 31th December 2008 (censoring information) or age at death as the dependent variable with a delayed entry date of 5th December 2000. To compare with a Gaussian linear regression approach we used an author programmed MLE function for censored Gaussian regression with left-truncated observations, not further described here. HMD data were analyzed using the same regression models but without delayed entry or censoring.

### Simulation study for model distribution misspecification

Parametric modeling assumes that a given underlying distribution function is the true distribution of the outcome variable. We performed a simulation study to assess whether life expectancy estimates of a skew-normal regression and a Gompertz survival regression are biased in case of model distribution misspecification. For that purpose we first fitted Gompertz and skew-normal models to Swiss data of the Human Mortality Database to get location, scale and shape parameters for each distribution, as close to real data as possible. Second, using these parameter estimates, we built random samples with different samples sizes (i.e., 100, 1,000, and 10,000) from Gompertz and skew-normal distribution functions. Third, for each sample we fitted a Gompertz or a skew-normal model and reported estimated life expectancy $\hat {\mu }$ and corresponding confidence intervals. As a third distribution function we combined the Gompertz and skew-normal distribution functions to get a mixture distribution functions with mixing proportions *δ* = {0.1, 0.25, 0.5, 0.75, 0.9}, where the mixing pdf is defined as *m*(*x*) = *δf_G_*(*x*) + (1 − *δ*) *g*(*x*) with *f_G_*, the Gompertz pdf, and *g*(*x*) the skew-normal pdf, defined as in Eq. (3). Thus, with the mixture distribution we mimic a situation where the study sample consists of samples from different underlying distribution functions, with different mixing proportions. For each sample we reported coverage of the true mean life expectancy *μ*_0_ and the bias $\hat {\mu }-{\mu }_{0}$. We did 1,000 simulation repetitions.

## Results

Results from parametric survival regression outputs using SNC data are summarized in the first part of Table 1. From a PH Weibull model, married women with a tertiary education (reference women) have a lower hazard of dying HR = 0.54, 95% CI [0.53–0.54], compared to married men with a tertiary education (reference men). Similar hazard ratios were obtained in a Gompertz survival model. The shape parameter from a Weibull regression model was estimated as 4.36, 95% CI [4.35–4.37], and from a Gompertz regression model as 0.104, 95% CI [0.104–0.105], thus both indicating evidence for a negative skewed distribution function. Results from AFT models lead to similar conclusions, but now expressed in time ratios, e.g., women have a 1.153, 95% CI [1.152–1.155], longer survival time compared to men in an AFT model with Weibull distribution, and a time ratio of 1.237, 95% CI [1.234–1.240] for a log-normal AFT model. Again we have evidence for a skewed distribution form. As mentioned above, parameter estimates from PH and AFT models are on a transformed scale and regression outputs are not in the metric of life expectancy. Results from Gaussian-type regression models are directly in terms of differences of mean life expectancy: Women tend to live on average 6.01, 95% CI [5.95–6.07], years longer than men using a censored skew-normal regression, compared to a difference of 6.66, 95% CI [6.59–6.74] years estimated in a Gaussian regression. The estimated shape parameter *γ* = − 0.782, 95% CI [−0.785–−0.779], from a skew-normal distribution indicated evidence for a negative skewed distribution.

**Table 1:** Regression output from different regression models using Swiss National Cohort data.

PH	Weibull^a^	[95% CI]	Gompertz^a^	[95% CI]
Reference: Male	1.00		1.00	
Female	0.54	[0.53, 0.54]	0.53	[0.53, 0.54]
Reference: Married	1.00		1.00	
Single	1.82	[1.80, 1.83]	1.62	[1.61, 1.64]
Widowed	1.73	[1.72, 1.75]	1.44	[1.43, 1.45]
Divorced	1.52	[1.51, 1.54]	1.52	[1.50, 1.53]
Reference: Tertiary	1.00		1.00	
Compulsory	1.46	[1.45, 1.48]	1.41	[1.40, 1.42]
Secondary	1.27	[1.25, 1.28]	1.26	[1.24, 1.27]
Not known	1.34	[1.31, 1.37]	1.17	[1.15, 1.20]
Location	–		–	
Scale *α*	53.83	[53.72, 53.94]	4.00e −4	[3.94e−4, 4.04e−4]
Shape *γ*	4.36	[4.35, 4.37]	0.104	[0.104, 0.105]

AFT	Weibull^b^	[95% CI]	Log-normal^b^	[95% CI]
Reference: Male	1.000		1.000	
Female	1.153	[1.152, 1.155]	1.237	[1.234, 1.240]
Reference: Married	1.000		1.000	
Single	0.872	[0.870, 0.874]	0.762	[0.759, 0.764]
Widowed	0.882	[0.880, 0.883]	0.677	[0.674, 0.680]
Divorced	0.908	[0.906, 0.910]	0.877	[0.874, 0.880]
Reference: Tertiary	1.000		1.000	
Compulsory	0.916	[0.914, 0.919]	0.846	[0.843, 0.849]
Secondary	0.947	[0.945, 0.950]	0.916	[0.913, 0.919]
Not known	0.936	[0.931, 0.940]	0.785	[0.778, 0.792]
Location	–		51.54	[51.40, 51.69]
Scale *α*	53.83	[53.72, 53.94]	0.424	[0.423, 0.425]
Shape *γ*	4.36	[4.35, 4.37]	–	

GT	Skew-normal (CP)^c^	[95% CI]	Gaussian^c^	[95% CI]
Reference: Male	0.00		0.00	
Female	6.01	[5.95, 6.07]	6.66	[6.59, 6.74]
Reference: Married	0.00		0.00	
Single	−4.95	[−5.04, −4.86]	−6.89	[−6.99, −6.78]
Widowed	−3.84	[−3.92, −3.76]	−5.85	[−5.96, −5.73]
Divorced	−3.99	[−4.10, −3.88]	−4.58	[−4.69, −4.47]
Reference: Tertiary	0.00		0.00	
Compulsory	−3.42	[−3.52, −3.32]	−3.74	[−3.85, −3.64]
Secondary	−2.25	[−2.34, −2.16]	−2.37	[−2.47, −2.28]
Not known	−1.96	[−2.17, −1.76]	−4.30	[−4.56, −4.03]
Location	47.96	[47.88, 48.05]	48.85	[48.76, 48.93]
Scale *α*	11.93	[11.90, 11.95]	12.87	[12.84, 12.90]
Shape *γ*	−0.782	[−0.785, −0.779]	–	–

**Notes.**

a Hazard ratios reported.

b Time ratios reported.

c Differences in life expectancy reported.

PH Proportional hazard model AFT Accelerated failure time model GT Gaussian-type CP Centered parametrization with reported skewness index *γ* CI Confidence interval

The upper part of Table 2 provides estimates of remaining life expectancy at age 35 years derived from parametric PH and AFT survival models using SNC data. Life expectancy at age 35 years for men ranged from 48.00 years (PH Gompertz model) to 56.39 years (AFT log-normal model), and ranged from 53.92 years (PH Gompertz model) to 69.77 years (AFT log-normal model) for women. Estimates from a censored skew-normal model were 47.96 years, and for a Gaussian model 48.85 years.

**Table 2:** Remaining life expectancy at age 35 years when analyzing Swiss National Cohort data.

**PH**	Weibull	[95% CI]	Gompertz	[95% CI]
Reference: Male				
Female	56.56	[56.42, 56.70]	53.92	[53.82, 54.02]
Reference: Married				
Single	42.76	[42.64, 42.87]	43.46	[43.35, 43.57]
Widowed	43.23	[43.12, 43.33]	44.61	[44.51, 44.71]
Divorced	44.52	[44.39, 44.66]	44.10	[43.98, 44.23]
Reference: Tertiary				
Compulsory	44.94	[44.87, 45.01]	44.78	[44.72, 44.84]
Secondary	46.46	[46.40, 46.52]	45.86	[45.81, 45.91]
Not known	45.88	[45.67, 46.09]	46.50	[46.31, 46.69]
Remaining life expectancy	49.04	[48.94, 49.13]	48.00	[47.92, 48.08]

**AFT**	Weibull	[95% CI]	Log-normal	[95% CI]
Reference: Male				
Female	56.56	[56.42, 56.70]	69.77	[69.51, 70.02]
Reference: Married				
Single	42.76	[42.64, 42.87]	42.95	[42.78, 43.13]
Widowed	43.23	[43.12, 43.33]	38.19	[38.02, 38.36]
Divorced	44.52	[44.39, 44.66]	49.46	[49.25, 49.68]
Reference: Tertiary				
Compulsory	44.94	[44.87, 45.01]	47.69	[47.57, 47.82]
Secondary	46.46	[46.40, 46.52]	51.64	[51.52, 51.75]
Not known	45.88	[45.67, 46.09]	44.24	[43.85, 44.62]
Remaining life expectancy	49.04	[48.94, 49.13]	56.39	[56.22, 56.56]

**GT**	Skew-normal	[95% CI]	Gaussian	[95% CI]
Reference: Male				
Female	53.97	[53.91, 54.03]	55.51	[55.44, 55.59]
Reference: Married				
Single	43.01	[42.92, 43.10]	41.96	[41.86, 42.07]
Widowed	44.12	[44.04, 44.20]	43.00	[43.89, 44.12]
Divorced	43.97	[43.86, 44.08]	44.27	[44.16, 44.38]
Reference: Tertiary				
Compulsory	44.54	[44.44, 44.64]	45.11	[45.00, 45.21]
Secondary	45.71	[45.62, 45.80]	46.48	[46.38, 46.57]
Not known	46.00	[45.79, 46.20]	44.55	[44.29, 44.82]
Remaining life expectancy	47.96	[47.88, 48.05]	48.85	[48.76, 48.93]

**Notes.**

PH PH Proportional hazard model AFT Accelerated failure time model GT Gaussian-type CI Confidence interval

Remaining life expectancy at age 35 years from HMD data are summarized in Table 3. Results from regression models are in the range 49.07 years (skew-normal model) to 50.60 (AFT log-normal model) years for women, and 45.01 years (skew-normal model) to 46.16 (AFT log-normal model) years for men. Note that the results for the Weibull distribution are identical in the PH and the AFT situation, as the models are mathematically equivalent. Remaining life expectancy at age 35 years from official estimates was 49.91 years for women and 45.69 years for men in 2008 (Human Mortality Database, 2014). Goodness-of-fit measured by *X*^2^ was lowest for a Gompertz survival model and the skew-normal regression model. Thus, both showed best model fit. Highest *X*^2^ were obtained for the AFT log-normal model and the Gaussian regression model, indicating worst model fit among all investigated models.

**Table 3:** Remaining life expectancy at age 35 years estimated from official death rates 2008 (simulated 100,000 individuals), by gender.

	RLE	[95% CI]	*X*^2^ (DF = 70)
**Women**			
PH Weibull	49.18	[49.11, 49.26]	6,020
PH Gompertz	49.56	[49.49, 49.64]	1,087
AFT Weibull	49.18	[49.11, 49.26]	6,020
AFT log-normal	50.60	[50.47, 50.73]	20,333
Skew-normal	49.07	[49.00, 49.13]	2,131
Gaussian	49.42	[49.35, 49.49]	9,441
*HMD estimate*	49.91		
**Men**			
PH Weibull	44.75	[44.67, 44.84]	6,339
PH Gompertz	45.31	[45.23, 45.40]	547
AFT Weibull	44.75	[44.67, 44.84]	6,339
AFT log-normal	46.16	[46.02, 46.30]	20,102
Skew-normal	45.01	[44.94, 45.08]	822
Gaussian	45.20	[45.12, 45.27]	7,810
*HMD estimate*	45.69		

**Notes.**

CI Confidence interval DF Degrees of freedom PH Proportional hazards model AFT Accelerated failure time model HMD Human Mortality Database RLE Remaining life expectancy

Coverage proportions and biases from the simulation study are reported in Table 4. We found that the coverage proportions of the Gompertz model and the skew-normal model were similar for small and moderate sample sizes, also when the underlying distribution function was misspecified. Coverage proportions were approximately 0.95. However, the Gompertz model showed a slightly smaller bias compared to the skew-normal model. For a larger sample size of 10,000 the skew-normal model showed worse coverage proportions compared to the Gompertz model (i.e., 0.72 if the true underlying distribution function was Gompertz). In case of a mixture distribution we found that the skew-normal model overestimates the true mean life expectancy with increasing mixture weights for Gompertz (*δ* = 0.1: −0.0166 ± 0.1080, *δ* = 0.5: −0.0433 ± 0.1114, *δ* = 0.9: −0.0747 ± 0.1189), leading to decreasing coverage proportions of (*δ* = 0.1: 0.946, *δ* = 0.5: 0.926, *δ* = 0.9: 0.868).

**Table 4:** Simulation study: coverage proportion and bias.

	*Model distribution*			
	Coverage proportion		Bias^a^ ± SD	
	Gompertz	Skew-normal	Gompertz	Skew-normal
*True underlying distribution* ^b^				
**Sample size 100**				
Skew-normal	0.946	0.940	0.0490 ± 1.1100	0.0620 ± 1.1040
Gompertz	0.959	0.938	0.0090 ± 1.0580	−0.1140 ± 1.1030
Mixture^c^				
*δ* = 0.1	0.952	0.945	0.0030 ± 1.1200	0.0070 ± 1.0900
*δ* = 0.25	0.951	0.946	−0.0110 ± 1.1180	−0.0200 ± 1.1020
*δ* = 0.5	0.950	0.944	−0.0190 ± 1.1170	−0.0460 ± 1.1130
*δ* = 0.75	0.952	0.943	−0.0150 ± 1.1090	−0.0550 ± 1.1140
*δ* = 0.9	0.951	0.940	−0.0040 ± 1.1130	−0.0590 ± 1.1240
**Sample size 1,000**				
Skew-normal	0.937	0.942	−0.0038 ± 0.3605	−0.0136 ± 0.3488
Gompertz	0.964	0.937	0.0282 ± 0.3312	−0.1160 ± 0.3505
Mixture^c^				
*δ* = 0.1	0.936	0.945	0.0120 ± 0.3620	−0.0010 ± 0.3500
*δ* = 0.25	0.945	0.949	0.0090 ± 0.3563	−0.0174 ± 0.3507
*δ* = 0.5	0.947	0.944	0.0004 ± 0.3558	−0.0428 ± 0.3545
*δ* = 0.75	0.943	0.937	−0.0002 ± 0.3573	−0.0599 ± 0.3594
*δ* = 0.9	0.943	0.932	0.0023 ± 0.3560	−0.0717 ± 0.3624
**Sample size 10,000**				
Skew-normal	0.959	0.955	0.0067 ± 0.1094	0.0019 ± 0.1065
Gompertz	0.963	0.715	−0.0020 ± 0.1038	−0.1470 ± 0.1108
Mixture^c^				
*δ* = 0.1	0.951	0.946	0.0037 ± 0.1104	−0.0166 ± 0.1080
*δ* = 0.25	0.954	0.935	0.0012 ± 0.1111	−0.0290 ± 0.1111
*δ* = 0.5	0.955	0.926	0.0022 ± 0.1089	−0.0433 ± 0.1114
*δ* = 0.75	0.953	0.898	0.0014 ± 0.1097	−0.0607 ± 0.1160
*δ* = 0.9	0.951	0.868	0.0013 ± 0.1099	−0.0747 ± 0.1189

**Notes.**

a Bias defined as the true underlying mean minus the estimated mean from Gompertz model or Skew-normal model.

b Used distribution parameters for Gompertz distribution: Shape parameter *γ* = 0.116, scale parameter *α* = exp − 12.25; For skew-normal distribution: Location parameter *μ* = 82.1, scale parameter *α* = 11.1, shape parameter *γ* = − 0.836.

c Mixture distribution: *δ* × Gompertz + (1 − *δ*) × Skew-normal.

SD Standard deviation

**Figure 1:**
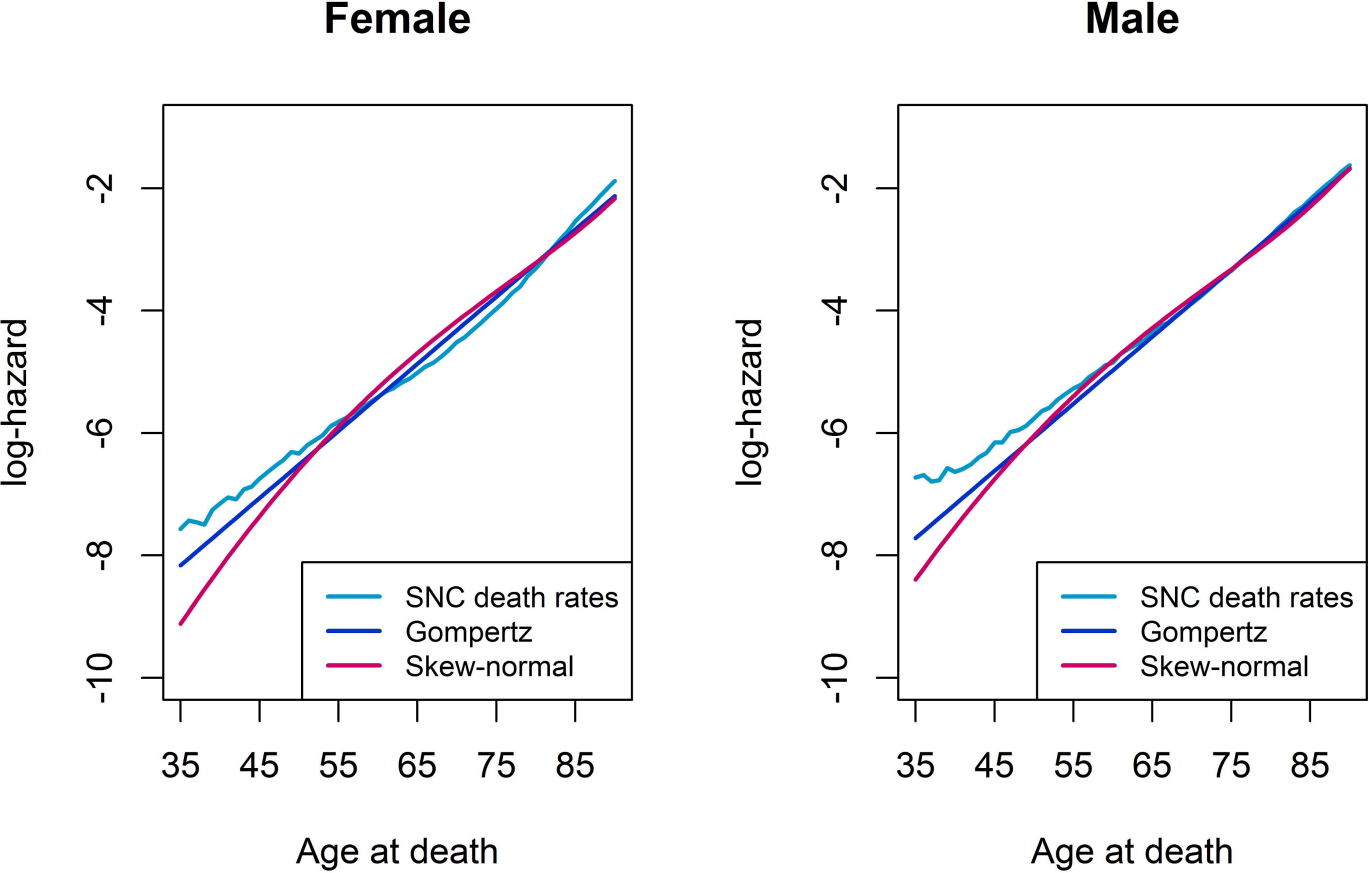
Log-hazard plots of SNC death rates, Gompertz proportional hazard model, and skew-normal model, by gender.

**Figure 2:**
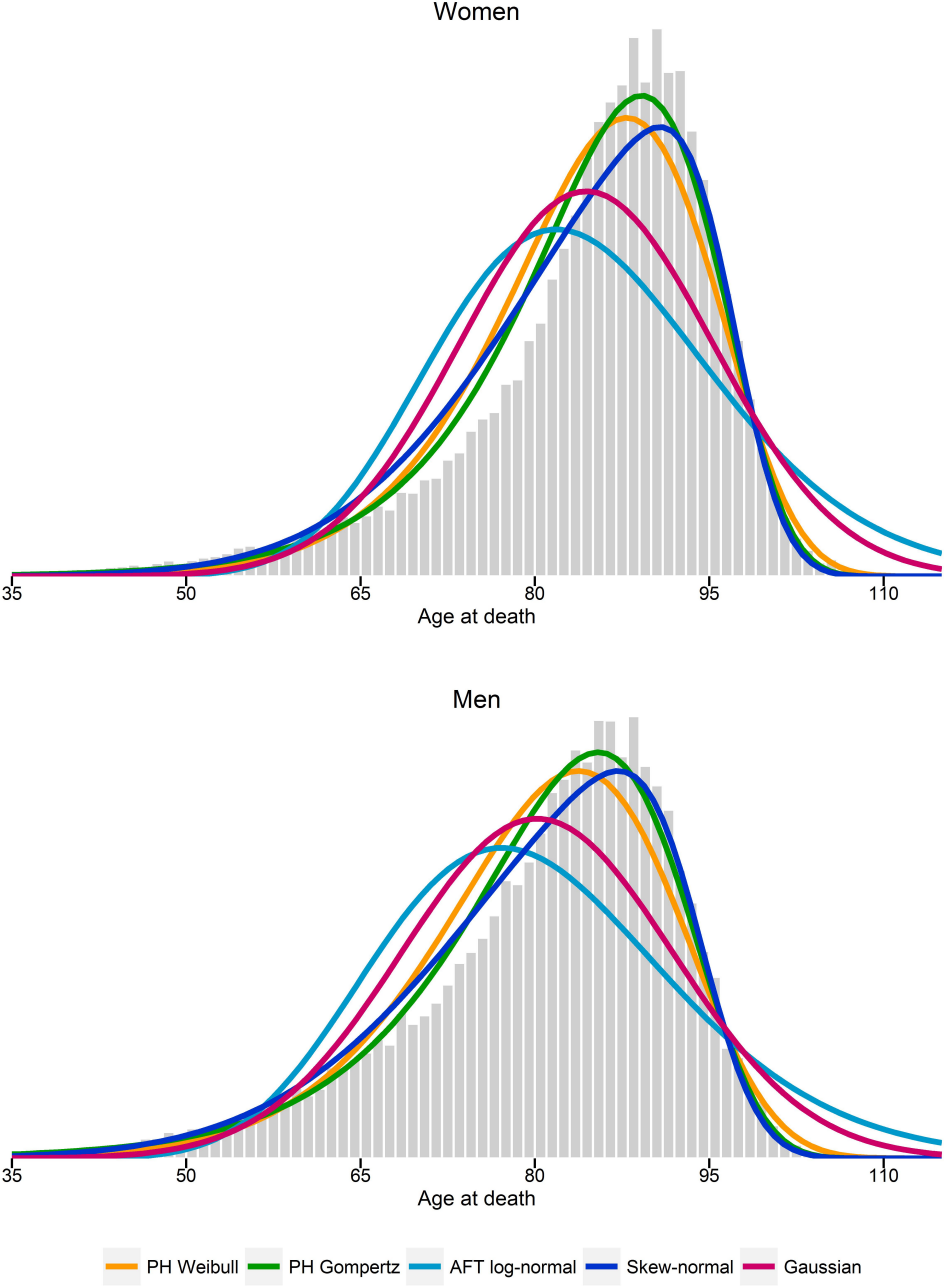
Histogram of estimated number of deaths per one-year age intervals. Probability density functions from estimated parameters from proportional hazard (PH) Weibull and Gompertz models, accelerated failure time (AFT) log-normal model, and skew-normal and Gaussian regression models.

Figure 1 compares the log-hazards from a Gompertz model (dark-blue line), a skew-normal model (red line) and from SNC data (light-blue line), by gender. Both a Gompertz and a skew-normal model underestimates the log-hazard at ages from 35 to 55. Nevertheless, the Gompertz model shows a slightly better model fit at these ages, compared to a skew-normal model. From age 50 onwards, a Gompertz model and a skew-normal model show almost identical model fits. Figure 2 shows a histogram of number of deaths per one year age intervals from the hypothetical population per gender. Density lines for each model were overlaid. Goodness-of-fit measured by *X*^2^ was lowest for a Gompertz survival model and the skew-normal regression model. Thus, both showed best model fit. Highest *X*^2^ were obtained for the AFT log-normal model and the Gaussian regression model, indicating worst model fit among all investigated models.

## Discussion

For modeling absolute differences in life expectancy, we compared results from commonly used parametric survival regression models to those obtained from a skew-normal regression model. We implemented a censored skew-normal regression approach which allowed to account for left-truncated observations having censored survival times. Our findings suggest that a censored skew-normal regression model is an adequate approach in the analysis of absolute differences in life expectancy. Surprisingly, statistical software like Stata (Stata Corporation, College Station, TX, USA) or R (R Project, University of Vienna, Austria) do not support skew-normal regression approaches in their core. A Stata suite for skew-normal and skew-*t* models has been introduced (Marchenko & Genton, 2010), and an R package for fitting univariate and multivariate skew-normal and skew-*t* models is available in the package sn (Azzalini, 2011). However, both available additions do not allow for regressions with left-truncated and censored observations.

Parametric survival models are often used to investigate the association of covariates on survival time. Effect measures are reported either as hazard ratios or time ratios, which yield no direct interpretation in terms of differences in survival time or life expectancy. Differences in life expectancy and corresponding confidence intervals from parametric survival models have to be calculated from estimated distributional and further model parameters from the underlying survival distribution function, using non-trivial transformations. Our presented skew-normal regression approach has the advantage that parameter estimates are directly interpretable in terms of absolute differences in life expectancy, similar to results from a linear Gaussian regression. In contrast to a Gaussian regression model, the negative skewness of lifetime data seemed reasonably captured when assuming a skew-normal distribution. Analyzing mortality data from the Swiss National Cohort or hypothetical data derived from official death rates in Switzerland showed that results, in terms of differences in life expectancy and goodness-of-fit, were comparable to those of commonly used parametric survival regression models, especially the Gompertz model commonly used for the analysis of life expectancy (Robertson, De Los Campos & Allison, 2013). Our results confirm the results from Robertson and colleagues (2012) that differences in life expectancy are similar across Gaussian-type regression models and parametric survival models, which can be explained by the central limit theorem. Our simulation study showed that the Gompertz model had better true mean life expectancy coverage in case of model misspecification compared to the skew-normal model. Thus, parameter estimates and standard errors from the skew-normal model could be more biased than those from a Gompertz model.

Our presented approach has limitations. First, the skew-normal distribution has a support on the real line ℝ, such that life expectancy could be estimated within an implausible range. Such situations could occur in a population with a high proportion of dead individuals compared to surviving individuals, and the individuals died very young. Then, most of the survival information lies in the upper end of the left-tail, and estimates from a skew-normal distribution could be implausible. Second, it is well-known that the distribution of time from birth to death has a bimodal shape, i.e., peaks occur at early infancy and older ages (Robertson & Allison, 2012). Our current approach does not include the modeling of bimodal distributed data, i.e., through mixtures of skew-normal distribution functions (Lin, Lee & Yen, 2007) or semiparametric approaches (Ma & Hart, 2007). Third, possible bias in the estimation of life expectancy is introduced by censoring. From the 4.1 mio investigated individuals in the SNC roughly 480,000 persons died over eight years of follow-up. Thus, 88.3% of the study population is censored and only 11.7% have exact time to death information. By study design this is a type-I censoring situation, and most of the likelihood information is driven by a pre-determined time point *c_i_* in the likelihood function defined in the survival function *S*(*c_i_*; *ξ*, *σ*^2^, *ψ*) in Eq. (6).

Besides the mentioned analysis methods described so far, other approaches of analysing mean survival time have been proposed. For example, Andersen and colleagues (2004) used so called pseudo-observations for the estimation of (restricted) mean survival time. Pseudo-observations are defined as “leave-one-out” estimators, i.e., parameters are estimated on subsamples where one observations is omitted, and is thus related to jackknife procedures. The advantage of this approach is that for the calculation of restricted mean survival time nonparametric estimators (i.e., Kaplan–Meier estimator), but also parametric survival models (i.e., using standard survival distribution functions) in the regression setting, can be used to calculate the pseudo-observations. Another approach is the use of flexible survival regression techniques (Royston & Lambert, 2011). Flexible survival regression models model the baseline cumulative hazard function using restricted cubic splines, and allow the calculations of restricted mean survival time (Royston & Parmar, 2013).

We conclude that a censored skew-normal regression approach is a possible alternative to parametric survival models for modeling differences in life expectancy. The advantage of this approach over parametric survival regression techniques is that parameter estimates are directly in terms of mean life expectancy. Other underlying Gaussian-type distribution functions (i.e., the skew-*t* distribution or the compressed Gaussian distribution) have been investigated (Clark et al., 2013; Robertson & Allison, 2012), with promising results in terms of model fit. In our analysis and simulation study the skew-normal distribution did not outperform the Gompertz distribution function. However, we found the skew-normal distribution a good compromise compared to other distribution functions in terms of model fit and modeling complexity. For example, fitting a skew-*t* distribution to larger data sets is computationally more intense due to the additional distributional complexity. Obviously a weighing of the gain in the use of more complex distribution function is needed, especially when differences in mean life expectancy are of main interest. A censored skew-normal regression approach is an alternative to existing Gaussian-type regression approaches in the modeling of life expectancy with the advantage of parameter estimates directly expressed in differences of life expectancy.

## Supplemental Information

10.7717/peerj.1162/supp-1 Supplemental Information 1 Supplemental Information

## Additional Information and Declarations

### Data Availability

The following information was supplied regarding the deposition of related data: The Swiss National Cohort is open to all researchers (http://www.swissnationalcohort.ch). Individual data from different data sets were used for the construction of the Swiss National Cohort. All these data are the property of the Swiss Federal Statistical Office (SFSO) and can only be made available by legal agreements with the SFSO. This also applies to derivatives such as the analysis files used for this study. However, after approval of the SNC Scientific Board, a specific SNC module contract with the SFSO would allow researchers to receive analysis files for replication of the analysis. Interested researchers can fill in the contact form on the homepage of the Swiss National Cohort (http://www.swissnationalcohort.ch/index.php?id=3002), or contact the Swiss National Cohort project managers by email (snc_info@ispm.unibe.ch).
