# Supplementary material for: Modeling absolute differences in life expectancy with a censored skew-normal regression approach
Source: PeerJ. 2015 Aug 6;3:e1162. doi: 10.7717/peerj.1162 (PMC4558072; doi:10.7717/peerj.1162)
Supplement: Supplemental Information 1 [file peerj-03-1162-s001.pdf]

SUPPLEMENTAL INFORMATION

Direct parametrization and centered parametrization

In the Methods section we described two possible parametrization of a skew-normal distributed random variable, the direct parametrization (DP) and the centered parametrization (CP). Here, we point out the principles and the conversion between the two parametrizations.

The DP has the disadvantage that singularity problems occur if $\psi = 0$ when estimating the parameters by maximum likelihood estimation (Azzalini, 1985). Another representation was then proposed by centering the parameters (Azzalini, 1985). The idea behind this centering is to reparametrize the problem by

$$Y = \mu + \alpha Z_0,$$

where Z_0 is constructed from a $Z \sim SN(0,1,\psi)$ random variable in the following way: For $\mu_z = E(Z) = \sqrt{\frac{2}{\pi}}\frac{\psi}{\sqrt{1+\psi^2}}$ and $\alpha_z = \sqrt{1-\mu_z^2}$ let

$$Z_0 = \frac{Z-\mu_z}{\alpha_z}.$$

By using equation (5) in the main manuscript, i.e.

$$\gamma = \frac{1}{2}(4-\pi)\text{sign}(\psi)\left(\frac{\psi^2}{\frac{\pi}{2}+\left(\frac{\pi}{2}-1\right)\psi^2}\right)^{3/2},$$

one obtains a measure of skewness. For the conversion of CP to DP the following formulae can be used (Genton, 2004),

$$\xi = \mu - \alpha\left(\frac{2\gamma}{4-\pi}\right)^{1/3}, \quad \sigma = \alpha\left[1+\left(\frac{2\gamma}{4-\pi}\right)^{2/3}\right]^{1/2},$$
$$\psi = \left(\frac{2\gamma}{4-\pi}\right)^{1/3}\left[\frac{2}{\pi}+\left(\frac{2\gamma}{4-\pi}\right)^{2/3}\left(\frac{2-\pi}{\pi}\right)\right]. \quad \text{(A1)}$$

Program code

In this section we provide the underlying program code for the statistical software R and Stata for the censored skew-normal regression problem with delayed entry. The derivation of the likelihood function is similar as described in (Azzalini and Capitanio, 1999) or (Genton, 2004) for uncensored observations.

We assume that a random variable Y is distributed as $SN(\mu,\alpha^2,\psi)$, which is equivalent to $Y = \mu + \alpha\left(\frac{Z-E(Z)}{\sqrt{Var(Z)}}\right) = \mu + \alpha Z_0$ with $Z \sim SN(0,1,\psi)$. Let D be the random variable indicating delayed entry. Note that (see e.g. Genton (2004))

$$E(Z) = \sqrt{\frac{2}{\pi}}\frac{\psi}{\sqrt{1+\psi^2}} \quad \text{and} \quad Var(Z) = 1-\frac{2}{\pi}\frac{\psi^2}{1+\psi^2}. \quad \text{(A2)}$$

We assume that each individual's age Y_i, $i \le n$, is identically and independently distributed as $SN(\mu,\alpha^2,\psi)$ and independent of censoring. Since $Z_i = E(Z_i) + \sqrt{Var(Z_i)}\left(\frac{Y_i-\mu}{\alpha}\right)$, a CP form of the likelihood function

in equation (6) in the main manuscript for left-truncated observations D_i is given as

$$\prod_{i\leq n}\left[\frac{\sqrt{Var(Z_i)}}{\alpha}\varphi\left(E(Z_i)+\sqrt{Var(Z_i)}\left(\frac{y_i-\mu}{\alpha}\right)\right)\right.$$
$$\Phi\left(\psi\left(E(z_i)+\sqrt{Var(Z_i)}\left(\frac{y_i-\mu}{\alpha}\right)\right)\right)$$
$$\left.\left[1-SN\left(E(Z_i)+\sqrt{Var(Z_i)}\left(\frac{d_i-\mu}{\alpha}\right);0,1,\psi\right)\right]^{-1}\right]^{\delta_i}$$
$$\left[\left(1-SN\left(E(Z_i)+\sqrt{Var(Z_i)}\left(\frac{y_i-\mu}{\alpha}\right);0,1,\psi\right)\right)\right.$$
$$\left.\left(\left[1-SN\left(E(Z_i)+\sqrt{Var(Z_i)}\left(\frac{d_i-\mu}{\alpha}\right);0,1,\psi\right)\right]^{-1}\right)\right]^{1-\delta_i}.$$

Since $E(Z_i)$ and $Var(Z_i)$ depend only on ψ and are independent of the index i, one obtains by using equations (A2) the following log-likelihood function for uncensored observations

$$\sum_{i\leq n:\ \delta_i=1}\log\left(\sqrt{1-\frac{2}{\pi}\frac{\psi^2}{1+\psi^2}}\right)-\log(\alpha)-$$
$$\frac{1}{2}\left(\sqrt{\frac{2}{\pi}}\frac{\psi}{\sqrt{1+\psi^2}}+\sqrt{1-\frac{2}{\pi}\frac{\psi^2}{1+\psi^2}}\left(\frac{y_i-\mu}{\alpha}\right)\right)^2+$$
$$\log\left(\Phi\left(\psi\left[\sqrt{\frac{2}{\pi}}\frac{\psi}{\sqrt{1+\psi^2}}+\sqrt{1-\frac{2}{\pi}\frac{\psi^2}{1+\psi^2}}\left(\frac{Y_i-\mu}{\alpha}\right)\right]\right)\right)-$$
$$\log\left(1-SN\left(\sqrt{\frac{2}{\pi}}\frac{\psi}{\sqrt{1+\psi^2}}+\sqrt{1-\frac{2}{\pi}\frac{\psi^2}{1+\psi^2}}\left(\frac{d_i-\mu}{\alpha}\right);0,1,\psi\right)\right), \qquad \text{(A3)}$$

and similarly for censored observations

$$\sum_{i\leq n:\ \delta_i=0}\log\left[1-SN\left(\sqrt{\frac{2}{\pi}}\frac{\psi}{\sqrt{1+\psi^2}}+\sqrt{1-\frac{2}{\pi}\frac{\psi^2}{1+\psi^2}}\left(\frac{y_i-\mu}{\alpha}\right);0,1,\psi\right)\right]$$
$$\log\left(1-SN\left(\sqrt{\frac{2}{\pi}}\frac{\psi}{\sqrt{1+\psi^2}}+\sqrt{1-\frac{2}{\pi}\frac{\psi^2}{1+\psi^2}}\left(\frac{d_i-\mu}{\alpha}\right);0,1,\psi\right)\right). \qquad \text{(A4)}$$

To get the skewness parameter γ one simply converts ψ by equation (5) in the main manuscript.

For MLE in R we first defined the negative likelihood function in a R function with five arguments `sn.lik.cen(param,y,X,cen,trun)`, where `param` are values for the vector of regression coefficients and the scale and shape parameters from the skew-normal distribution α and ψ given in (A3) or (A4). `y` corresponds to the dependent variable, `X` the model matrix of covariates, `cen` is an indicator for censored observations, and `trun` is the variable for delayed entry.

```
sn.lik.cen <- function(param,y,X,cen,trun) {

 k <- ncol(X)
 beta <- param[1:k]
 alpha <- param[k+1]
 psi <- param[k+2]

 # Residual vectors
 e <- y-X\%*\%beta
 truncen <- trun-X\%*\%beta
```

```
 # Log likelihood of uncensored observations
 logl_uncen <- (1-cen)*(
  log(sqrt(1-2/pi*psi^2/(1+psi^2))/alpha)
   -0.5*(sqrt(2/pi)*psi/sqrt(1+psi^2)
    +sqrt(1-2/pi*psi^2/(1+psi^2))*(e/alpha))^2
  +log(pnorm(psi*(sqrt(2/pi)*psi/sqrt(1+alpha^2)
    +sqrt(1-2/pi*psi^2/(1+psi^2))*(e/alpha))))
  -log(1-psn(sqrt(2/pi)*alpha/sqrt(1+psi^2)
    +sqrt(1-2/pi*psi^2/(1+psi^2))*(truncen/alpha),
     location=0, scale=1, shape=psi))
 )

 # Log likelihood of censored observations
 logl_cen <- cen*(
  log(1-psn(sqrt(2/pi)*alpha/sqrt(1+psi^2)
   +sqrt(1-2/pi*psi^2/(1+psi^2))*(e/alpha),
    location=0, scale=1, shape=psi)
  -log(1-psn(sqrt(2/pi)*alpha/sqrt(1+psi^2)
   +sqrt(1-2/pi*psi^2/(1+psi^2))*(truncen/alpha),
    location=0, scale=1, shape=psi))
 )

 # Return negative log likelihood function
 return(-sum(logl_uncen+logl_cen))
}
```

Within this function we used the probability density function `psn` from a skew-normal distribution from the R package `sn` (Azzalini, 2011). In second step we maximized the negative log likelihood function by the `optim` function available within the R core `stats` library, using a quasi-Newton algorithm (option `method="BFGS"` within the `optim` command).

For MLE in Stata we first defined the likelihood five parameter function `sn` with parameters: `logl`, the log likelihood function; `mu`, the location parameter used for centering the dependent variable by the linear combination of covariates $\mathbf{X}^{\top}\beta$; `alph` and `psi`, the scale and shape parameter from the skew-normal distribution α and ψ given in (A3) or (A4). Censored observations and the variable for delayed entry are indicated by the `local censor` and `local ltrun` macro. No cdf program function exists in Stata for a skew-normal distribution. We used a conditioning method on a bivariate normal cdf (see e.g. Genton (2004), Proposition 1.2.2) to get the corresponding cdf of a skew-normal distribution. In the second step the log likelihood function was maximized by the `ml model lf [...], technique(nr)` command using a Newton-Raphson algorithm, followed by `ml max` to obtain the parameter estimates. `ml` places the name of a dependent variable in a global macro, denoting internally the dependent variable as `$ML_y1`. The likelihood function is defined with the `program define` command as follows.

```
program define sn_cen

 args logl mu alph psi

 local censor "$S_cen"
 local ltrun "$S_ltrun"

 quietly replace `logl'=cond( `censor'==0,

 * `censor'==0: Log likelihood of uncensored observations
 ln(sqrt(1-2/pi*`psi'^2/(1+`psi'^2))/`alph')
  -0.5*(sqrt(2/pi)*`psi'/sqrt(1+`psi'^2)
   +sqrt(1-2/pi*`psi'^2/(1+`psi'^2))
    *(($ML_y1-`mu')/`alph'))^2
```

```
 +ln(normal(`psi'*(sqrt(2/pi)*`psi'/sqrt(1+`psi'^2)
  +sqrt(1-2/pi*`psi'^2/(1+`psi'^2))
   *(($ML_y1-`mu')/`alph'))))
 -ln(1-2*(binormal(0,sqrt(2/pi)*`psi'/sqrt(1+`psi'^2)
  +((`ltrun'-`mu')/`alph')
   *sqrt(1-2/pi*`psi'^2/(1+`psi'^2)),
    -`psi'/( sqrt(1+`psi'^2) )))),

 * `censor'==1: Log likelihood of censored observations
 ln(1-2*(binormal(0,sqrt(2/pi)*`psi'/sqrt(1+`psi'^2) ///
  +sqrt(1-2/pi*`psi'^2/(1+`psi'^2))
   *(($ML_y1-`mu')/`alph'),
    -`psi'/( sqrt(1+`psi'^2) ))))
 -ln(1-2*(binormal(0,sqrt(2/pi)*`psi'/sqrt(1+`psi'^2)
  +((`ltrun'-`mu')/`alph')
   *sqrt(1-2/pi*`psi'^2/(1+`psi'^2)),
    -`psi'/( sqrt(1+`psi'^2) ))))
 )
end
```

There could be situations where the MLE has convergence problems, mainly in data sets with small to moderate sample size. This problem has been described (Azzalini and Capitanio, 1999) and could be solved by an EM algorithm. In larger data sets, especially the analyzed data within this manuscript, this situation did not occur.

We compared the output from our own written program codes with the results of the available MLE function for skew-normal regression for the uncensored case. In R we used within the package `sn` the function `sn.mle()` (Azzalini, 2011). In Stata we used the `skewnreg` function from the available suite for skew-normal regression (Marchenko and Genton, 2010). All obtained results were similar up to negligible numerical approximation differences.

REFERENCES

Azzalini, A. (1985). A class of distribution which includes the normal ones. *Scand J Statist*, 12(2):171–178.

Azzalini, A. (2011). *R package sn: The skew-normal and skew-t distributions (Version 0.4-17)*.

Azzalini, A. and Capitanio, A. (1999). Statistical applications of the multivariate skew normal distribution. *J R Stat Soc, Ser B, Stat Methodol*, 61(3):579–602.

Genton, M. G., editor (2004). *Skew-elliptical distibutions and their applications: A journey beyond normality*. Chapman & Hall/CRC, Boca Raton, FL.

Marchenko, Y. V. and Genton, M. G. (2010). A suite of commands for fitting the skew-normal and skew-t models. *The Stata Journal*, 10(4):507–539.
